# Neurodegeneration and humoral response proteins in cerebrospinal fluid associate with pediatric-onset multiple sclerosis and not monophasic demyelinating syndromes in childhood

**DOI:** 10.1177/13524585221125369

**Published:** 2022-09-24

**Authors:** Arlette L Bruijstens, Christoph Stingl, Coşkun Güzel, Marcel P Stoop, Yu Yi M Wong, E Daniëlle van Pelt, Brenda L Banwell, Amit Bar-Or, Theo M Luider, Rinze F Neuteboom, Dutch Pediatric MS and ADEM Study Group; Canadian Pediatric Demyelinating Disease Network

**Affiliations:** Department of Neurology, Erasmus University Medical Center, Rotterdam, The Netherlands; Laboratory of Neuro-Oncology, Clinical and Cancer Proteomics, Department of Neurology, Erasmus University Medical Center, Rotterdam, The Netherlands; Laboratory of Neuro-Oncology, Clinical and Cancer Proteomics, Department of Neurology, Erasmus University Medical Center, Rotterdam, The Netherlands; Laboratory of Neuro-Oncology, Clinical and Cancer Proteomics, Department of Neurology, Erasmus University Medical Center, Rotterdam, The Netherlands; Department of Neurology, Erasmus University Medical Center, Rotterdam, The Netherlands; Department of Neurology, Erasmus University Medical Center, Rotterdam, The Netherlands; Division of Child Neurology, Department of Neurology, The Children’s Hospital of Philadelphia, Perelman School of Medicine, University of Pennsylvania, Philadelphia, PA, USA; Center for Neuroinflammation and Experimental Therapeutics and Division of Multiple Sclerosis and Related Disorders, Department of Neurology, Perelman Center for Advanced Medicine (PCAM), University of Pennsylvania; Laboratory of Neuro-Oncology, Clinical and Cancer Proteomics, Department of Neurology, Erasmus University Medical Center, Rotterdam, The Netherlands; Department of Neurology, Erasmus University Medical Center, Rotterdam, The Netherlands

**Keywords:** Cerebrospinal fluid, proteins, proteomics, mass spectrometry, neurons, immune system, pediatrics, multiple sclerosis

## Abstract

**Background:**

Pediatric-onset multiple sclerosis (POMS) represents the earliest stage of disease pathogenesis. Investigating the cerebrospinal fluid (CSF) proteome in POMS may provide novel insights into early MS processes.

**Objective:**

To analyze CSF obtained from children at time of initial central nervous system (CNS) acquired demyelinating syndrome (ADS), to compare CSF proteome of those subsequently ascertained as having POMS versus monophasic acquired demyelinating syndrome (mADS).

**Methods:**

Patients were selected from two prospective pediatric ADS studies. Liquid chromatography–mass spectrometry (LC-MS) was performed in a Dutch discovery cohort (POMS *n* = 28; mADS *n* = 39). Parallel reaction monitoring–mass spectrometry (PRM-MS) was performed on selected proteins more abundant in POMS in a combined Dutch and Canadian validation cohort (POMS *n* = 48; mADS *n* = 106).

**Results:**

Discovery identified 5580 peptides belonging to 576 proteins; 58 proteins were differentially abundant with ⩾2 peptides between POMS and mADS, of which 28 more abundant in POMS. Fourteen had increased abundance in POMS with ⩾8 unique peptides. Five selected proteins were all confirmed within validation. Adjusted for age, 2 out of 5 proteins remained more abundant in POMS, that is, *Carboxypeptidase E (CPE)* and *Semaphorin-7A (SEMA7A)*.

**Conclusion:**

This exploratory study identified several CSF proteins associated with POMS and not mADS, potentially reflecting neurodegeneration, compensatory neuroprotection, and humoral response in POMS. The proteins associated with POMS highly correlated with age at CSF sampling.

## Introduction

Despite extensive research, the exact pathogenesis of MS remains to be elucidated. Substantial (indirect) evidence indicates that biological start of disease likely substantially precedes first clinical symptoms, potentially with onset during childhood even when disease does not manifest until years into adulthood.^1,2^ However, in 3%–5% of all MS patients, first clinical symptoms occur in childhood.^3^ Therefore, pediatric-onset multiple sclerosis (POMS) provides a unique opportunity to gain key insights into an early stage of the disease. As cerebrospinal fluid (CSF) potentially reflects processes occurring within the central nervous system (CNS),^4^ proteins in CSF of POMS patients might reveal crucial information about very early MS disease biology.

Previously, one Dutch and one Canadian study have focused on CSF proteins in POMS.^5,6^ Due to the rarity of POMS, these studies included a limited number of patients. The aim of current collaborative study was to explore and validate differences in the CSF proteome in a larger, combined Dutch and Canadian cohort of children with an initial CNS acquired demyelinating syndrome (ADS), comparing those subsequently ascertained as having either POMS or monophasic ADS (mADS) .

## Materials and methods

### Patients and samples

Patients were selected from two independent, prospective observational cohort studies including ADS patients <18 years: the Dutch PROUD-kids study (*PRedicting the OUtcome of a first Demyelinating event in childhood*)^7^ and Canadian Pediatric Demyelinating Disease Study.^8^ Patients with available CSF samples collected at incident demyelinating attack were included if, at final follow-up, patients were diagnosed with (1) POMS or (2) mADS, according to International Pediatric MS Study Group criteria.^9^ Demographic, clinical, and laboratory data collected in both prospective cohort studies were used.

**Figure 1.**
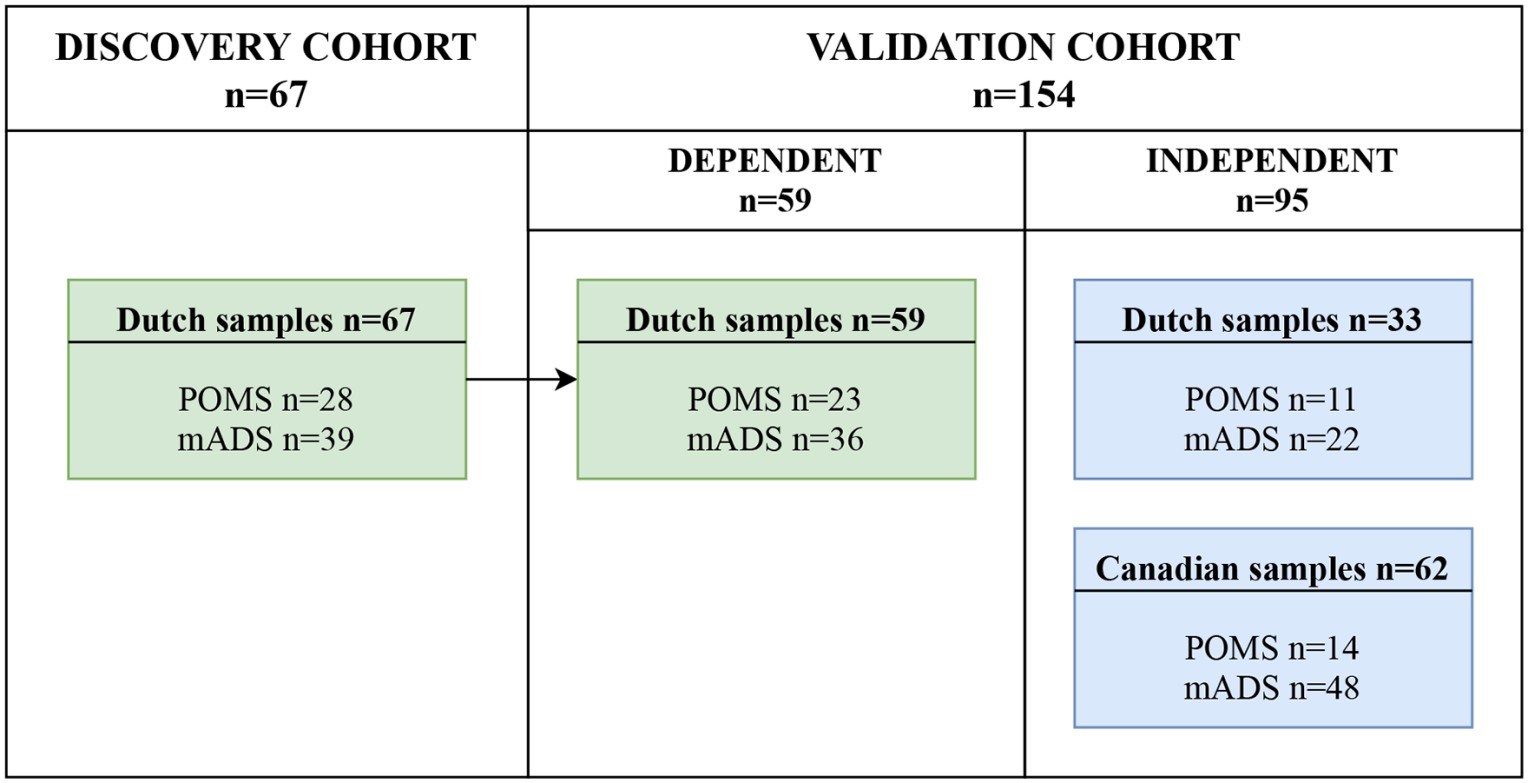
Sample selection of discovery and validation cohort. mADS: monophasic acquired demyelinating syndrome; POMS: pediatric-onset multiple sclerosis.

For this study, we used samples from the previously performed Dutch^5^ and Canadian^6^ proteomic studies, supplemented by new samples. Figure 1 shows the sample selection for this study. For the untargeted discovery analysis, only Dutch samples were selected (discovery cohort). For the following targeted validation analysis, both dependent (from discovery analysis) and independent (newly acquired) samples were selected (validation cohort). The dependent samples were used for technical validation regarding the different techniques used for discovery and validation. The independent samples consisted of Canadian and newly acquired Dutch samples. CSF samples of adult symptomatic controls (*n* = 16), having neurological symptoms but no objective (para)clinical findings to define a specific neurological disease,^10^ were pooled for technical quality control (QC) in validation analysis.

### Ethical approval and patients consents

Protocols of the Dutch and Canadian prospective cohort study were approved by the local Medical Ethical Committee, and all patients and/or their legal representatives gave written informed consent.

### Discovery analysis

#### Liquid chromatography–mass spectrometry (LC-MS)

For discovery analysis, CSF proteins were typically digested and prepared.^11^ Subsequently, digested samples were measured on a nano-liquid chromatography–Orbitrap Fusion mass spectrometer liquid chromatography-mass spectrometry, and the data were subsequently processed in a fragment mass spectrometer database search and label-free quantitative analysis.^12^ A detailed description and adjustments with regard to cited references are provided in Supplemental Material S1. Liquid chromatography-mass spectrometry (LC-MS) data have been deposited to the ProteomeXchange Consortium via the PRIDE^13^ partner repository with dataset identifier PXD031004 and 10.6019/PXD031004.

#### Statistical analysis of discovery proteomics

The normalized abundances on all individual peptides were compared between POMS and mADS by performing a Wilcoxon rank-sum test. Proteins were deemed to be significantly differentially abundant between the groups if they were identified by two or more peptides and passed a set of three separate stringent criteria: (1) At least 25% of the peptides of the protein had a very low *p* value (*p* < 0.01); (2) at least 50% of the peptides of the protein had a low *p* value (*p* < 0.05); and (3) at least 75% of the peptides of the protein were altered in the same direction between groups (i.e. increased or decreased abundance in POMS), with a slight modification to criteria published previously.^14^ False discovery rate (FDR) of the detection of significant proteins was determined by performing the statistical analysis 50 times on permutated datasets, whereby for each permutation, the samples were assigned randomly to a category.

### Validation analysis

#### Protein selection

After discovery analysis, proteins identified more abundant in POMS with eight or more peptides belonging to one protein were considered for validation. Five proteins were selected for validation based on (1) their potential function (CNS or immune related) and (2) their established significant fold change in the discovery analysis. Proteins with both low- and high-fold changes were selected to cover the complete fold-change range. Selected proteins were validated in the validation cohort (Figure 1), using parallel reaction monitoring-mass spectrometry (PRM-MS).

#### Parallel reaction monitoring-mass spectrometry (PRM-MS)

Validation analysis was conducted as previously described,^12^ with small alterations. Details of sample preparations and data acquisition are reported in Supplemental Material S1 and information on the set of target peptides in Supplemental Table S2. PRM-MS data have been deposited to the ProteomeXchange Consortium via the PRIDE^13^ partner repository with dataset identifier PXD031022 and 10.6019/PXD031022. The PRM-MS signals were integrated using Skyline software.^15^

#### Statistical analysis of patient characteristic and validation proteomics

SPSS software version 25.0 was used, with a significance level of *p* < 0.05. Demographic data were compared between POMS and mADS groups. Chi-square (or Fisher’s exact) and Student’s t-test (or Wilcoxon rank sum test) were used when appropriate for categorical and continuous data, respectively. Because protein concentrations expressed by PRM-MS ratios were neither normally distributed, nor after log-transformation, non-parametric tests were used. Comparison of PRM-MS ratios between groups was performed with Wilcoxon rank sum test. Correlations were studied using Spearman’s rank correlation coefficient. Effects of covariates on PRM-MS ratios were assessed by linear regression.

## Results

### Patient characteristics

The discovery cohort consisted of 67 Dutch patients (POMS *n* = 28, mADS *n* = 39; Figure 1). In 59 patients, remaining CSF samples were available for further analyses. Subsequently, these 59 dependent samples were used in the validation cohort to enable technical validation. In addition, 95 independent newly acquired CSF samples (33 Dutch and 62 Canadian) were selected for the validation cohort, resulting in a total validation cohort of 154 patients (POMS *n* = 48, mADS *n* = 106; Figure 1).

Table 1 shows patient characteristics of the discovery and validation cohort, the latter divided into dependent and independent samples. In the dependent and independent validation samples, the same differences were observed; patients in the POMS group compared with the mADS group were older (14.6 vs. 6.4 and 14.5 vs. 8.4 years for the dependent and independent validation samples, respectively, both *p* < 0.001), more often had unique oligoclonal bands (OCBs) in CSF (100% vs. 13% and 96% vs. 12%, respectively, both *p* < 0.001), and had longer time between onset of disease and lumbar puncture (22.0 vs. 12.0 and 28.0 vs. 4.5 days, respectively, both *p* < 0.001). The mADS group compared with POMS group more often included patients with acute disseminated encephalomyelitis (ADEM; 61% vs. 0% and 40% vs. 0%, respectively, both *p* < 0.001) and more often included myelin oligodendrocyte glycoprotein antibody (MOG-ab) seropositive patients (27% vs. 0% (*p* = 0.018) and 33% vs. 0% (*p* = 0.001), respectively), tested by cell-based assay. Seven Dutch patients included in the POMS group had an unknown or inconclusive MOG-ab status; however, none of these patients was qualified as typical MOG-ab associated disorder; six patients had a typical clinical and radiological MS disease course, and one patient was diagnosed with tumefactive MS. This latter patient had negative aquaporin-4 antibody testing and an inconclusive MOG-ab test (both by cell-based assay). CSF examination in this patient showed a high IgG index and presence of OCB. Besides OCBs, other CSF parameters available and follow-up duration were not different between groups. Finally, comparing POMS and mADS separated for Dutch and Canadian validation samples showed the same results (Supplemental Table S3).

**Table 1.** Patient characteristics of the samples used in the discovery and validation cohort.

	Discovery cohort			Validation cohort					
				Dependent samples			Independent samples		
	POMS *n* = 28	mADS *n* = 39	*p* value*	POMS *n* = 23	mADS *n* = 36	*p* value*	POMS *n* = 25	mADS *n* = 70	*p* value*
Sex, no of females (%)	16 (57)	24 (62)	NS	13 (57)	21 (58)	NS	15 (60)	28 (40)	NS
Age at onset, y, median (IQR)	14.9 (13.5–16.0)	5.8 (3.2–10.8)	**<0.001**	14.6 (13.3–15.9)	6.4 (3.0–10.9)	**<0.001**	14.5 (13.6–15.3)	8.4 (4.5–12.2)	**<0.001**
ADEM, *n* (%)	0	25 (64)	**<0.001**	0	22 (61)	**<0.001**	0	28 (40)	**<0.001**
Blood analyses									
MOG-ab seropositive, *n* (%)	0/22	9/35 (26)	**0.009**	0/19	9/33 (27)	**0.018**	0/24	23/69 (33)	**0.001**
AQP4-ab seropositive, *n* (%)	0/11	0/18	na	0/8	0/17	na	0/23	0/66	na
CSF analyses									
RBC, median (IQR)	0.0 (0.0–207.0)	0.0 (0.0–12.0)	NS	0.5 (0.0–53.0)	0.0 (0.0–9.0)	NS	0.0 (0.0-–2.0)	0.0 (0.0–8.0)	NS
WBC, median (IQR)	8.0 (5.0–28.0)	8.0 (3.0–41.3)	NS	11.5 (5.0–28.3)	7.0 (3.0–37.0)	NS	8.0 (4.0–20.5)	9.0 (2.1–32.0)	NS
Total protein, median (IQR)	0.32 (0.25–0.37)	0.29 (0.22–0.43)	NS	0.33 (0.25–0.38)	0.28 (0.21–0.42)	NS	0.34 (0.27–0.41)	0.27 (0.22–0.40)	NS
Unique OCBs, *n* (%)	25/26 (96)	4/32 (13)	**<0.001**	22/22 (100)	4/30 (13)	**<0.001**	24/25 (96)	6/62 (12)	**<0.001**
Time between disease onset and LP, d, median (IQR)	23.0 (9.25–68.5)	11.0 (5.0–20.0)	**0.007**	22.0 (9.0–71.0)	12.0 (5.0–21.5)	**0.026**	28.0 (5.0–43.5)	4.5 (3.0–12.8)	**<0.001**
DMT use at time of LP (%)	0	0	na	0	0	na	0	0	na
FU, m, median (IQR)	67.5 (46.3–98.0)	87.0 (40.0–107.0)	NS	62.0 (45.0–98.0)	75.5 (40.3–109.3)	NS	36.0 (25.5–109.0)	56.5 (17.3–96.0)	NS

* POMS vs. mADS using chi-square (Fisher’s exact) or Wilcoxon rank-sum test.

ADEM: acute disseminated encephalomyelitis; AQP4-ab: aquaporin-4 antibody; CSF: cerebrospinal fluid; DMT: disease modifying therapy; FU: follow-up; IQR: interquartile range; LP: lumbar puncture; mADS: monophasic acquired demyelinating syndrome; MOG-ab: myelin oligodendrocyte glycoprotein antibody; na: not applicable; OCBs: oligoclonal bands; POMS: pediatric-onset multiple sclerosis; RBC: red blood cell count; WBC: white blood cell count.

### Discovery of proteins with LC-MS

Using LC-MS in CSF samples of the discovery cohort (*n* = 67), a total of 5580 peptides was identified, belonging to 576 proteins; 58 proteins were found with two or more peptides significantly different between POMS and mADS (FDR 4.8%; Table 2), of which 28 with increased abundance in POMS.

**Table 2.** Discovery analysis—Identification of CSF proteins differentially abundant POMS (*n* = 28) and pediatric mADS (*n* = 39).

Direction of difference in POMS	#	Accession	Gene	Protein description	Fold change^a^	# of **peptides**^b^
**Increased abundance**	1	P01599	IGKV1-17	Immunoglobulin kappa variable 1-17	5.168	7
	2	P06331	IGHV4-34	Immunoglobulin heavy variable 4-34	4.768	3
	3	P01611	IGKV1D-12	Immunoglobulin kappa variable 1D-12	4.362	4
	**4**	**P06312**	**IGKV4-1**	**Immunoglobulin kappa variable 4-1**	**4.267**	**10**
	5	P06310	IGKV2-30	Immunoglobulin kappa variable 2-30	4.14	4
	6	P01615	IGKV2D-28	Immunoglobulin kappa variable 2D-28	3.342	6
	**7**	**P01593**	**IGKV1D-33**	**Immunoglobulin kappa variable 1D-33**	**3.284**	**14**
	8	P01824	IGHV4-39	Immunoglobulin heavy variable 4-39	2.656	2
	9	P01594	IGKV1-33	Immunoglobulin kappa variable 1-33	2.368	4
	**10**	**P01834**	**IGKC**	**Immunoglobulin kappa constant**	**2.092**	**12**
	**11**	**P01857**	**IGHG1**	**Immunoglobulin heavy constant gamma 1**	**2.06**	**25**
	**12**	**P01859**	**IGHG2**	**Immunoglobulin heavy constant gamma 2**	**1.834**	**25**
	13	P01763	IGHV3-48	Immunoglobulin heavy variable 3-48	1.76	4
	**14**	**O75326**	**SEMA7A**	**Semaphorin-7A**	**1.736**	**8**
	15	P04430	IGKV1-16	Immunoglobulin kappa variable 1-16	1.637	4
	**16**	**Q96KN2**	**CNDP1**	**Beta-Ala-His dipeptidase**	**1.553**	**25**
	17	P20933	AGA	*N*(4)-(beta-N-acetylglucosaminyl)-L-asparaginase	1.516	3
	18	P34096	RNASE4	Ribonuclease 4	1.509	2
	**19**	**P16870**	**CPE**	**Carboxypeptidase E**	**1.463**	**13**
	**20**	**Q7Z7M0**	**MEGF8**	**Multiple epidermal growth factor-like domains protein 8**	**1.438**	**13**
	21	Q06828	FMOD	Fibromodulin	1.39	3
	**22**	**Q92876**	**KLK6**	**Kallikrein-6**	**1.386**	**11**
	23	P26992	CNTFR	Ciliary neurotrophic factor receptor subunit alpha	1.341	3
	**24**	**Q96** **GW7**	**BCAN**	**Brevican core protein**	**1.329**	**14**
	**25**	**Q7Z3B1**	**NEGR1**	**Neuronal growth regulator 1**	**1.318**	**8**
	**26**	**Q02818**	**NUCB1**	**Nucleobindin-1**	**1.282**	**9**
	**27**	**Q9UBP4**	**DKK3**	**Dickkopf-related protein 3**	**1.232**	**18**
	28	P80748	IGLV3-21	Immunoglobulin lambda variable 3-21	1.17	4
**Decreased abundance**	29	P18669	PGAM1	Phosphoglycerate mutase 1	0.847	5
	30	P01023	A2M	Alpha-2-macroglobulin	0.823	109
	31	O43866	CD5 L	CD5 antigen-like	0.798	8
	32	P01024	C3	Complement C3	0.748	147
	33	P04217	A1BG	Alpha-1B-glycoprotein	0.712	22
	34	Q96IY4	CPB2	Carboxypeptidase B2	0.684	3
	35	P00450	CP	Ceruloplasmin	0.682	65
	36	P10643	C7	Complement component C7	0.644	27
	37	Q06033	ITIH3	Inter-alpha-trypsin inhibitor heavy chain H3	0.617	9
	38	P23083	IGHV1-2	Immunoglobulin heavy variable 1-2	0.598	2
	39	P02788	LTF	Lactotransferrin	0.596	4
	40	P62937	PPIA	Peptidyl-prolyl cis-trans isomerase A	0.568	8
	41	Q86VB7	CD163	Scavenger receptor cysteine-rich type 1 protein M130	0.559	16
	42	Q9Y6R7	FCGBP	IgGFc-binding protein	0.533	55
	43	P80723	BASP1	Brain acid soluble protein 1	0.522	10
	44	P02763	ORM1	Alpha-1-acid glycoprotein 1	0.517	15
	45	P00739	HPR	Haptoglobin-related protein	0.482	20
	46	P59665	DEFA1	Neutrophil defensin 1	0.445	2
	47	O75015	FCGR3B	Low-affinity immunoglobulin gamma Fc region receptor III-B	0.425	2
**Decreased abundance**	48	P00738	HP	Haptoglobin	0.42	34
	49	P62328	TMSB4X	Thymosin beta-4	0.395	6
	50	P29966	MARCKS	Myristoylated alanine-rich C-kinase substrate	0.387	2
	51	P31946	YWHAB	14-3-3 protein beta/alpha	0.371	2
	52	P61626	LYZ	Lysozyme C	0.346	4
	53	Q08ET2	SIGLEC14	Sialic acid-binding Ig-like lectin 14	0.346	4
	54	P10153	RNASE2	Non-secretory ribonuclease	0.328	2
	55	P08637	FCGR3A	Low-affinity immunoglobulin gamma Fc region receptor III-A	0.305	2
	56	Q8TEU8	WFIKKN2	WAP, Kazal, immunoglobulin, Kunitz, and NTR domain-containing protein 2	0.289	8
	57	Q9Y279	VSIG4	V-set and immunoglobulin domain-containing protein 4	0.195	4
	58	P48539	PCP4	Calmodulin regulator protein PCP4	0.121	2

Proteins listed in table were identified differentially abundant between POMS and mADS with at least two unique peptides significantly different in discovery analysis. In bold, the proteins with increased abundance in POMS with at least eight unique peptides were significantly different.

a Fold change > 1 = increased abundance in POMS. Fold change < 1 = decreased abundance in POMS.

b Number of differentially identified peptides for the same protein.

### Selection of proteins for validation

Proteins with increased abundance in POMS with eight or more peptides significantly different were considered for validation (Table 2; in bold). Based on function and significant fold change, the five proteins selected for validation included *SEMA7A, CPE, Multiple epidermal growth factor-like domains protein 8 (MEGF8), Neuronal growth regulator 1 (NEGR1)*, and *Nucleobindin-1 (NUCB1)* (Table 3). Immunoglobulins (Igs) were not selected for validation, as their role in POMS diagnosis is already established.^16,17^

**Table 3.** Proteins selected for validation.

Direction of difference in POMS	#	Accession	Gene	Protein description	Fold change^a^	No. of **peptides**^b^
Increased abundance	1	O75326	SEMA7A	Semaphorin-7A	1.736	8
	2	P16870	CPE	Carboxypeptidase E	1.463	13
	3	Q7Z7M0	MEGF8	Multiple epidermal growth factor-like domains protein 8	1.438	13
	4	Q7Z3B1	NEGR1	Neuronal growth regulator 1	1.318	8
	5	Q02818	NUCB1	Nucleobindin-1	1282	9

Proteins listed in table were proteins with increased abundance in POMS with eight or more peptides significantly different belonging to one protein in discovery analyses, and finally selected for validation based on their function (CNS or immune related) and their fold change (both low and high ranges).

a Fold change > 1 = increased abundance in POMS.

b Number of differentially identified peptides for the same protein.

### Validation of proteins with PRM-MS

**Figure 2.**
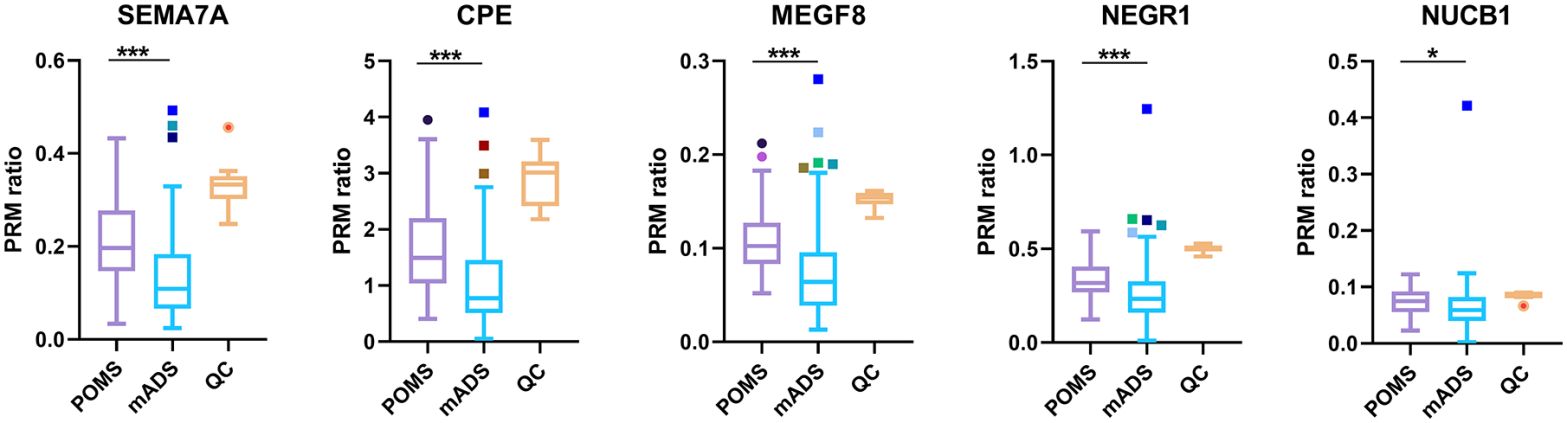
PRM-MS ratios of selected proteins for validation with PRM-MS measurements in POMS vs. mADS. CSF samples of adult controls (*n* = 16) were pooled and included for technical QC. Using Wilcoxon rank sum test; *p < 0.05, **p < 0.01, and ***p < 0.001. CPE: Carboxypeptidase E; mADS: monophasic acquired demyelinating syndrome; MEGF8: Multiple epidermal growth factor-like domains protein 8; NEGR1: Neuronal growth regulator 1; NUCB1: Nucleobindin-1; POMS: pediatric-onset multiple sclerosis; SEMA7A: Semaphorin-7A; QC: quality control.

Using PRM-MS, in the total validation cohort (*n* = 154), all five selected proteins were confirmed more abundant in POMS (*p* < 0.001 for *SEMA7A, CPE, MEGF8*, and *NEGR1; p* = 0.020 for *NUCB1*; Figure 2). In the dependent validation samples (technical validation), all proteins were confirmed more abundant in POMS. In the independent validation samples, four of five proteins were confirmed more abundant in POMS (*NUCB1* not). Analyzing the Dutch (*n* = 92) and Canadian (*n* = 62) samples of the total validation cohort separately, again four of five proteins were confirmed more abundant in POMS in both groups (*NUCB1* not).

Further sub-analyses of the five selected proteins were performed within the total validation cohort in order to utilize all acquired quantitative PRM-MS data.

### Effect of clinical phenotype and age

**Figure 3.**
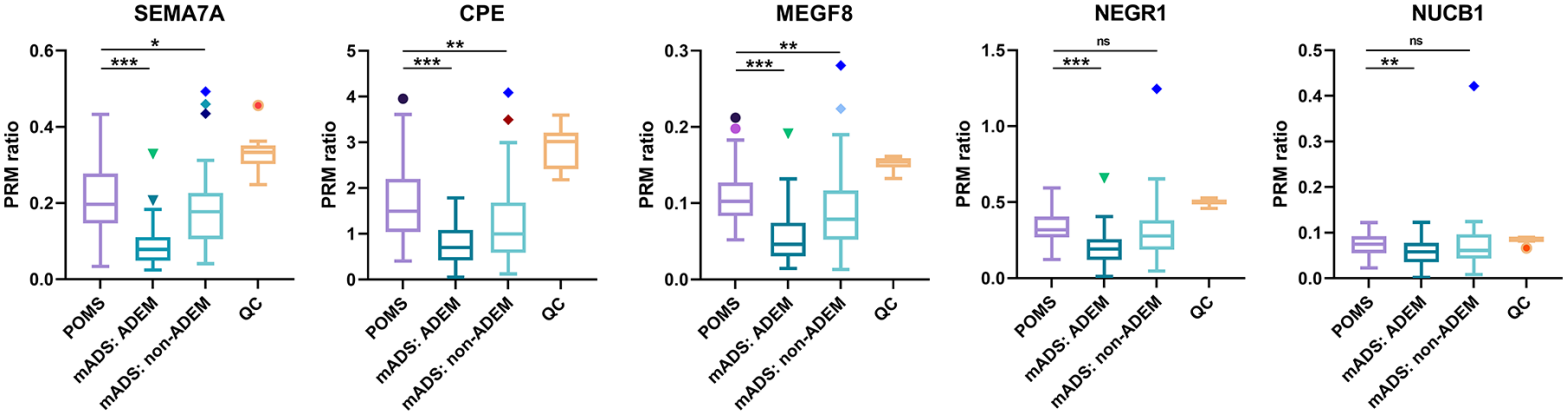
PRM-MS ratios of selected proteins for validation with PRM-MS measurements separated for clinical phenotype within mADS group in ADEM and non-ADEM patients. CSF samples of adult controls (*n* = 16) were pooled and included for technical QC. Using Wilcoxon rank sum test: *p < 0.05, **p < 0.01, and ***p < 0.001. ADEM: acute disseminated encephalomyelitis; CPE: Carboxypeptidase E; mADS: monophasic acquired demyelinating syndrome; MEGF8: Multiple epidermal growth factor-like domains protein 8; NEGR1: Neuronal growth regulator 1; NUCB1: Nucleobindin-1; POMS: pediatric-onset multiple sclerosis; SEMA7A: Semaphorin-7A; QC: quality control.

Some overlap was observed in PRM-MS ratios of POMS and mADS groups (Figure 2), but as described above, comparing the groups still resulted in significant differences. Further sub-analyses in the total validation cohort showed a clear distinction within the mADS group based on clinical phenotype, with lower PRM-MS ratios in mADS patients with an ADEM phenotype and higher PRM-MS ratios in mADS patients with a non-ADEM phenotype (Figure 3). However, compared with mADS non-ADEM group, *SEMA7A* (*p* = 0.047), *CPE* (*p* = 0.004), and *MEGF8* (*p* = 0.006) remained significantly more abundant in POMS.

Because mADS non-ADEM patients were older than mADS ADEM patients (11.1 vs. 4.6 years, *p* < 0.001), we hypothesized that age could contribute to observed differences in PRM-MS ratios. A significant positive correlation was found between age and PRM-MS ratio in the complete validation cohort, but also within mADS group (Spearman’s ρ ranging between 0.203 and 0.541 for all selected proteins) and mADS non-ADEM group (Spearman’s ρ ranging between 0.284 and 0.404 for all selected proteins except for *NUCB1*; Supplemental Table S4). Subsequent linear regression analysis showed that all selected proteins were significantly dependent on increasing age when adjusted for diagnosis (POMS vs. mADS). Conversely, proteins *SEMA7A* and *CPE* remained significantly more abundant in POMS compared with mADS when adjusted for age (*SEMA7A*: Canadian samples *p* = 0.041; *CPE*: complete validation cohort *p* = 0.036 and Canadian samples *p* = 0.018).

## Discussion

This exploratory collaborative study discovered and validated potentially interesting CSF proteins that may be involved in POMS and not mADS. Discovery analysis (LC-MS) with use of stringent statistical criteria resulted in a total of 58 identified proteins with at least two unique peptides differentially abundant between POMS and mADS, of which 28 with increased abundance in POMS. Applying even more stringent criteria, selecting only the proteins with at least eight unique peptides different identified 14 proteins with increased abundance in POMS (Table 2; in bold). A selection of five of these proteins, covering the entire fold-change range of discovery analysis, was made for subsequent validation with a high-resolution targeted quantitative proteomic approach (PRM-MS).^18^ All these five proteins were confirmed in the dependent validation samples and overall validation cohort, including the proteins with lowest fold-change ranges in the discovery analysis (e.g. *NUCB1* and *NEGR1*), indicating that our discovery analysis was already accurate, possibly due to the use of very stringent criteria. Therefore, the complete list of those 14 proteins may include potentially promising proteins involved in POMS.

Among these 14 promising proteins are Igs, which are a hallmark of MS disease and have been implemented in most recent MS diagnostic criteria in the form of oligoclonal Ig gamma (IgG) bands.^16^ Also, the kappa subtype of Ig has been studied as a potential biomarker for MS diagnosis and alternative for OCB.^19^ Two of five implicated Igs have been previously associated with POMS, namely *Ig heavy constant gamma 1*^5^ and *Ig heavy constant gamma 2*.^6^ These Igs underline the key role of the humoral immune system in POMS. The last implicated protein with an immunological function is *SEMA7A*, known to be involved in T-cell-mediated inflammation, and associated with more inflammatory lesions and demyelination in MS and experimental autoimmune encephalomyelitis.^20^

Our two previously conducted exploratory studies in POMS reported an increased abundance of predominantly gray matter^5^ and axoglial^6^ proteins in CSF. Similar to findings in these two studies,^5,6^ the remainder of the 14 promising proteins we implicate here in POMS mainly has neuron-related functions, including *Brevican core protein (BCAN), NEGR1, SEMA7A, CPE*, and *NUCB1. BCAN* inhibits neurite outgrowth,^21^ while *NEGR1* promotes neuronal growth.^22,23^ *SEMA7A*, in addition to its above-described immune-related function, is also expressed in the CNS by different (injured) neuronal and glial cells.^24^ *CPE* is a protease catalyzing a wide range of (neuro)peptides which are involved in neuronal structure and survival,^25^ and finally, *NUCB1* is exclusively found in neurons.^26^ Both *CPE* and *NUCB1* are reported to be upregulated after (oxidative) stress.^25,27^

We speculate that the multiple neuron-related proteins we implicate here in POMS versus mADS may reflect a combination of neurodegenerative and potentially compensatory neuroprotective mechanisms manifesting in MS but not in monophasic CNS inflammatory conditions. Neurodegeneration is considered also in other studies to be involved in POMS.^28,29^ Due to the ongoing CNS damage, compensatory neuroprotective pathways may have been initiated. In contrast, in mADS patients (in whom clinical and biological onset of the single event likely coincide), neurodegeneration may not be an ongoing process (or may not yet have been triggered), and hence, compensatory neuroprotective pathways are not initiated.

Three of these five neuron-related proteins have been reported previously with increased abundance in MS, of which *CPE*^5,6^ and *NEGR*^5^ only in POMS, and *BCAN* both in POMS^5^ and adult-onset multiple sclerosis (AOMS).^30^ Strikingly, *SEMA7A* was observed in lower concentrations in adults with clinically isolated syndromes (CIS) subsequently ascertained as having clinically definite MS versus CIS patients who remained monophasic during multiple years of follow-up,^31^ while in our study, we found an increased abundance in POMS versus mADS. Although our study does not include a direct AOMS CSF proteomic comparison, the fact that currently implicated proteins with potentially neuroprotective functions are reported mainly in POMS and less in AOMS, if confirmed, could indicate that repair and/or compensatory mechanisms might be superior in POMS compared with AOMS. This would be in line with other evidence that POMS patients exhibit less physical disability in the first-decade post-onset and have longer time to disease progression in spite of having increased inflammatory activity.^32^ Finally, protein *NUCB1* has never been described in MS, but interestingly, it has been associated with primary neurodegenerative disorders.^33^ However, as this protein was not confirmed significantly (only as a trend) in our independent validation samples, further studies are needed to determine whether there is a role of this protein in POMS.

Remaining proteins of the complete list of 14 include *Kallikrein-6* (*KLK6*), *Dickkopf-related protein 3* (*DKK3*), *MEGF8*, and *Beta-Ala-His dipeptidase*. Oligodendrocyte-derived *KLK-6* has been studied quite extensively in the context of MS (models) and is observed to be elevated in CSF of AOMS^34^ and POMS.^5^ Finally, *DKK3*^5^ and *MEGF8*^6^ were previously also found more abundant in POMS.

In the quantification analysis, it was possible to study the contribution of factors other than diagnosis on the concentrations of specific proteins. The clinical phenotype was found to be related to protein concentrations. However, age at time of CSF sampling was revealed to be the main contributing factor, as only *SEMA7A* and *CPE* remained significantly increased in POMS when corrected for age, substantiating the need for correction for age.

This study has several advantages and limitations. First, CSF remains the closest compartment to the CNS tissue which is still reasonably accessible. As the CSF composition may reflect the processes occurring in the diseased MS CNS,^4^ the analysis of the CSF proteome may provide unique insights into disease biology. Although it should be recognized that one is only indirectly (and likely only partially) sampling such processes. Second, although our sample size remains relatively limited, the current combination of samples from previously conducted studies^5,6^ with the addition of new samples increased sample size substantially and enabled both validation of multiple proteins (the overlap has been described above in detail) and extension of previous results in a combined cohort of pediatric ADS patients from separate countries. Third, the use of mADS as representative of other CNS inflammatory disorders serves as an excellent control group to identify CSF proteins specific for MS.^10^ Although our study has multiple years of follow-up (Table 1), future relapses in mADS patients who are MOG-ab seropositive cannot be ruled out, as a small proportion of these patients might experience new disease activity years after disease onset.^35^ Studying samples from age-matched healthy children would also be of interest, particularly to determine the extent to which observed differences between POMS and mADS CSF profiles may be driven by age-related changes in pediatric CSF composition. However, obtaining pediatric healthy control CSF samples is extremely difficult for ethical reasons, and CSF collected from healthy adults is not adequate for this purpose. Fourth, we focused on proteins with increased abundance in POMS, while proteins with decreased abundance in POMS could also be interesting in terms of disease biology. We decided to select five proteins more abundant in POMS for validation, which covered the complete fold-change range, in order to explore the accuracy of the LC-MS measurements, and we conclude that the remaining of the 14 proteins found in the discovery analysis are worth validating in an independent cohort as well.

In conclusion, this collaborative study found several CSF proteins with expected immunological but also neuronal functions to be involved in POMS, representing the earliest stage of MS that can be evaluated. These proteins provide potential insights into the underlying biology of POMS regarding neurodegeneration and possible compensatory neuroprotection. Besides our current confirmation of increased *SEMA7A* and *CPE* in POMS CSF, validation of remaining potentially interesting proteins is needed to determine their possible role after adjusting for age, as we note that differences in CSF proteins between pediatric patient cohorts are strongly age-associated. Further international collaborative efforts will be critical to enable both protein validation and additional discovery in this rare but relevant group of pediatric patients. Finally, a direct comparison between the CSF proteome of AOMS and POMS is of interest to confirm potential differences in pathobiology of early versus later-onset MS.

## Supplemental Material

sj-docx-1-msj-10.1177_13524585221125369 – Supplemental material for Neurodegeneration and humoral response proteins in cerebrospinal fluid associate with pediatric-onset multiple sclerosis and not monophasic demyelinating syndromes in childhood Click here for additional data file. Supplemental material, sj-docx-1-msj-10.1177_13524585221125369 for Neurodegeneration and humoral response proteins in cerebrospinal fluid associate with pediatric-onset multiple sclerosis and not monophasic demyelinating syndromes in childhood by Arlette L Bruijstens, Christoph Stingl, Coşkun Güzel, Marcel P Stoop, Yu Yi M Wong, E Daniëlle van Pelt, Brenda L Banwell, Amit Bar-Or, Theo M Luider and Rinze F Neuteboom in Multiple Sclerosis Journal

sj-docx-2-msj-10.1177_13524585221125369 – Supplemental material for Neurodegeneration and humoral response proteins in cerebrospinal fluid associate with pediatric-onset multiple sclerosis and not monophasic demyelinating syndromes in childhood Click here for additional data file. Supplemental material, sj-docx-2-msj-10.1177_13524585221125369 for Neurodegeneration and humoral response proteins in cerebrospinal fluid associate with pediatric-onset multiple sclerosis and not monophasic demyelinating syndromes in childhood by Arlette L Bruijstens, Christoph Stingl, Coşkun Güzel, Marcel P Stoop, Yu Yi M Wong, E Daniëlle van Pelt, Brenda L Banwell, Amit Bar-Or, Theo M Luider and Rinze F Neuteboom in Multiple Sclerosis Journal

sj-docx-3-msj-10.1177_13524585221125369 – Supplemental material for Neurodegeneration and humoral response proteins in cerebrospinal fluid associate with pediatric-onset multiple sclerosis and not monophasic demyelinating syndromes in childhood Click here for additional data file. Supplemental material, sj-docx-3-msj-10.1177_13524585221125369 for Neurodegeneration and humoral response proteins in cerebrospinal fluid associate with pediatric-onset multiple sclerosis and not monophasic demyelinating syndromes in childhood by Arlette L Bruijstens, Christoph Stingl, Coşkun Güzel, Marcel P Stoop, Yu Yi M Wong, E Daniëlle van Pelt, Brenda L Banwell, Amit Bar-Or, Theo M Luider and Rinze F Neuteboom in Multiple Sclerosis Journal

sj-docx-4-msj-10.1177_13524585221125369 – Supplemental material for Neurodegeneration and humoral response proteins in cerebrospinal fluid associate with pediatric-onset multiple sclerosis and not monophasic demyelinating syndromes in childhood Click here for additional data file. Supplemental material, sj-docx-4-msj-10.1177_13524585221125369 for Neurodegeneration and humoral response proteins in cerebrospinal fluid associate with pediatric-onset multiple sclerosis and not monophasic demyelinating syndromes in childhood by Arlette L Bruijstens, Christoph Stingl, Coşkun Güzel, Marcel P Stoop, Yu Yi M Wong, E Daniëlle van Pelt, Brenda L Banwell, Amit Bar-Or, Theo M Luider and Rinze F Neuteboom in Multiple Sclerosis Journal

sj-docx-5-msj-10.1177_13524585221125369 – Supplemental material for Neurodegeneration and humoral response proteins in cerebrospinal fluid associate with pediatric-onset multiple sclerosis and not monophasic demyelinating syndromes in childhood Click here for additional data file. Supplemental material, sj-docx-5-msj-10.1177_13524585221125369 for Neurodegeneration and humoral response proteins in cerebrospinal fluid associate with pediatric-onset multiple sclerosis and not monophasic demyelinating syndromes in childhood by Arlette L Bruijstens, Christoph Stingl, Coşkun Güzel, Marcel P Stoop, Yu Yi M Wong, E Daniëlle van Pelt, Brenda L Banwell, Amit Bar-Or, Theo M Luider and Rinze F Neuteboom in Multiple Sclerosis Journal
