# Supplementary material for: Neurodegeneration and humoral response proteins in cerebrospinal fluid associate with pediatric-onset multiple sclerosis and not monophasic demyelinating syndromes in childhood
Source: Mult Scler. 2022 Sep 24;29(1):52–62. doi: 10.1177/13524585221125369 (PMC9896265; doi:10.1177/13524585221125369)
Supplement: sj-docx-1-msj-10.1177_13524585221125369 – Supplemental material for Neurodegeneration and humoral response proteins in cerebrospinal fluid associate with pediatric-onset multiple sclerosis and not monophasic demyelinating syndromes in childhood [file sj-docx-1-msj-10.1177_13524585221125369.docx]

**Supplementary file S1- Proteomic sample preparation and data acquisition and analysis.**

*Discovery analysis with liquid chromatography-mass spectrometry (LC-MS))*

Discovery of possible relevant CSF proteins was performed with liquid chromatography-mass spectrometry (LC-MS). CSF samples (20 µL) of patients included in the discovery cohort were digested according to an in-solution trypsin digestion protocol, as described previously (11). Prepared samples were analysed on an Ultimate 3000 nano LC system online coupled to an Orbitrap Fusion mass spectrometer (Thermo Fischer Scientific, Germany), using a data-dependent acquisition method. Samples injected (3 µL digest) were transferred onto a trap column (C18 PepMap, 300 µm ID x 5 mm; Thermo Fisher Scientific) using 0.1% trifluoroacetic acid at a flow rate of 20 µl/min, and further eluted and separated on a 50 cm analytical nano-LC column (PepMap C18, 75 µm ID x 500 mm, 2 µm, 100 Å; Thermo Fisher Scientific) using a binary gradient from 4% to 38% B in 90 minutes, whereby solvent A was 0.1% formic acid, and solvent B 80% acetonitrile and 0.08% formic acid. Column flow rate was 300 nL/min and column temperature was 40 °C. For electrospray ionization we used coated silica nano electro-spray emitters (New Objective, Woburn, MA, USA) at a spray voltage of 1.8 kV. A data dependent acquisition MS method was used with an Orbitrap survey scan (range 375 - 1550 m/z, resolution of 120,000, AGC target 400,000), followed by consecutively isolation (isolation with of 1.6 amu), fragmentation (HCD, 28% normalized collision energy) and detection (ion trap, AGC target 10,000) of the peptide precursors detected in the survey scan until a duty cycle time of 3 seconds was exceeded. Dynamic exclusion was used with 10 ppm mass tolerance and 60 seconds exclusion duration.

Fragment mass spectra were searched against the human subset of Uniprot database (version 2015-11-12; 20,194 entries) using Mascot (version 2.3.02; Matrix Science, UK) using following parameters: proteolytic cleavage by trypsin, two missed cleavages allowed, oxidation of Met as variable modification and carbamidomethylation of Cys as fixed modification, precursor tolerance of 10 ppm and fragment tolerance of 0.5 u. Search results were post-processed with the software package Scaffold (version 3.6.3, Proteome Software, Portland, OR) to merge the individual search results, conduct protein grouping and calculate protein and peptide identification confidence levels (filter set to false discover rate <1%). For label-free quantification analysis, Progenesis LC-MS software package (version 4.0, Nonlinear Dynamics, Newcastle-upon-Tyne, Waters, United Kingdom) were used.

*Validation analysis with parallel reaction monitoring-mass spectrometry (PRM-MS)*

Ten μL of each CSF sample and QC pool was added to 47 μL digestion buffer containing 100 mM TEAB, 1% SDC, 10% Acetonitrile using a 96-well plate (Axygen 96-well plate, [Corning Life Sciences](https://de.vwr.com/store/supplier/id/AXYG/corning-life-sciences), VWR, Amsterdam, The Netherlands). A concentration of 37.5 fmol of a mixture of SIL peptides (Pepscan, Lelystad, The Netherlands; final 0.5 fmol/µl) was spiked and digested overnight by the addition of 400 ng trypsin (Promega, gold-grade) and incubation at 37°C with gentle shaking. Digests were stopped and detergent was precipitated by addition of 60 µL 1.5% TFA. The detergent was removed by centrifugation at 4,400 g and subsequently filtered through a 0.45 µm membrane (AcroPrep, PALL Laboratory, VWR, Amsterdam, The Netherlands) and transferred to a 384 well plate in two aliquots of 40 µL. The liquid handling was carried out in a BRAVO platform (Agilent Technologies, Waldbronn, Germany). PRM measurements were acquired on a nano‐LC system (Thermo Fisher Scientific, Germering, Germany) online coupled to an Orbitrap Fusion Lumos mass spectrometer (Thermo Fisher Scientific, San Jose, CA, US). Ten µL digest was loaded on a C18 trap column (C18 PepMap, 300 µm inner diameter (ID) × 5 mm, 5 µm particle size, 100 Å pore size; Thermo Fisher Scientific, The Netherlands) and desalted for 10 minutes using a flow rate of 20 µL/min 0.1% TFA. The trap column was switched online with the analytical column (PepMap C18, 75 µm ID ×250 mm, 2 µm particle, and 100 Å pore size; Thermo Fisher Scientific), and peptides were eluted with the following binary (A and B) gradient: 4%–38% solvent B in 30 minutes whereby solvent A consists of 0.1% formic in water and solvent B consists of 80% acetonitrile and 0.08% formic acid in water. The column flow rate was set to 300 nL/min. For electrospray ionization, nano ESI emitters (New Objective, Woburn, MA) were used, and a spray voltage of 2.0 kV was applied. Peptide specific settings of the PRM method are listed in Table S2. The targeted fragment mass spectrometry mode set up was used as follows: isolation width 1.0 Da, HCD fragmentation at optimized collision energy, ion injection time was set to 118 ms and AGC target to 50,000 ions, Orbitrap resolution of 60,000. The selection of the precursor ions was time scheduled, and each duty cycle consisted of six targeted fragment mass spectrometry scans (endogenous and SIL precursor of three peptides). Within each duty cycle, as well as an Orbitrap mass spectrometry survey scan from 350–1,400 Da with a resolution of 120,000 was carried out. Samples were distributed over two well plates and 15 measurements of the QC pool were conducted over the course of the sequence. PRM data was acquired and ratios between the endogenous and SIL peptides were calculated to determine the protein concentration. The PRM signals were integrated using Skyline software (15).
