# Supplementary material for: Neurodegeneration and humoral response proteins in cerebrospinal fluid associate with pediatric-onset multiple sclerosis and not monophasic demyelinating syndromes in childhood
Source: Mult Scler. 2022 Sep 24;29(1):52–62. doi: 10.1177/13524585221125369 (PMC9896265; doi:10.1177/13524585221125369)
Supplement: sj-docx-2-msj-10.1177_13524585221125369 – Supplemental material for Neurodegeneration and humoral response proteins in cerebrospinal fluid associate with pediatric-onset multiple sclerosis and not monophasic demyelinating syndromes in childhood [file sj-docx-2-msj-10.1177_13524585221125369.docx]

**Supplementary file S2.** Table with selected peptide specific settings of the PRM method^$^.

| **Gene** | **Peptide sequence** | **z** | **CE** | **m/z** | **t start** | **t stop** |
| --- | --- | --- | --- | --- | --- | --- |
| NUCB1 | LSQETEALGR | 2 | 30 | 552.2882 | 0.00 | 8.36 |
| NUCB1 | LSQETEALGR# | 2 | 30 | 557.2923 | 0.00 | 8.36 |
| NUCB1 | ELQQAVLHM*EQR | 3 | 37 | 499.9208 | 5.84 | 8.78 |
| NUCB1 | ELQQAVLHM*EQR# | 3 | 37 | 503.2569 | 5.84 | 8.78 |
| NEGR1 | SSIIFAGGDK | 2 | 22 | 497.7638 | 8.36 | 11.53 |
| NEGR1 | SSIIFAGGDK# | 2 | 22 | 501.7709 | 8.36 | 11.53 |
| NUCB1 | ELQQAVLHMEQR | 3 | 37 | 494.5892 | 8.78 | 12.50 |
| NUCB1 | ELQQAVLHMEQR# | 3 | 37 | 497.9252 | 8.78 | 12.50 |
| MEGF8 | FLDTGVVQSDR | 2 | 25 | 618.8146 | 9.45 | 12.72 |
| MEGF8 | FLDTGVVQSDR# | 2 | 25 | 623.8187 | 9.45 | 12.72 |
| CPE | IHIM*PSLNPDGFEK | 3 | 24 | 538.6046 | 11.53 | 13.90 |
| CPE | IHIM*PSLNPDGFEK# | 3 | 24 | 541.2760 | 11.53 | 13.90 |
| SEMA7A | GESELYTSDTVM*QNPQFIK | 3 | 23 | 735.0123 | 12.72 | 15.06 |
| SEMA7A | GESELYTSDTVM*QNPQFIK# | 3 | 23 | 737.6837 | 12.72 | 15.06 |
| CPE | IHIMPSLNPDGFEK | 3 | 24 | 533.2729 | 13.90 | 16.04 |
| CPE | IHIMPSLNPDGFEK# | 3 | 24 | 535.9443 | 13.90 | 16.04 |
| CPE | AASQPGELKDWFVGR | 3 | 38 | 554.2862 | 15.06 | 17.55 |
| CPE | AASQPGELKDWFVGR# | 3 | 38 | 557.6223 | 15.06 | 17.55 |
| SEMA7A | GESELYTSDTVMQNPQFIK | 3 | 23 | 729.6807 | 16.04 | 19.60 |
| SEMA7A | GESELYTSDTVMQNPQFIK# | 3 | 23 | 732.3521 | 16.04 | 19.60 |
| NEGR1 | VVVNFAPTIQEIK | 2 | 22 | 729.4218 | 16.36 | 32.00 |
| NEGR1 | VVVNFAPTIQEIK# | 2 | 22 | 733.4289 | 16.36 | 32.00 |

Gene: NUCB1 = Nucleobindin-1, NEGR1 = Neuronal growth regulator 1, MEGF8 = Multiple epidermal growth factor-like domains protein 8, CPE = Carboxypeptidase E, SEMA7A = Semaphorin-7A; Peptide sequence: asterisk (*) marks oxidation of Methionine; hash (#) indicates stable isotope labelled Lysine (L-Lysine-^13^C_6_, ^15^N_2_) or Arginine (L-Arginine-^13^C_6_, ^15^N_4_), respectively; z: charge state measured; CE: optimal normalized collision energy of HCD fragmentation; m/z: mass-over-charge ratio used; t(start) and t(stop): margins of scheduled PRM time windows.

^$^ Peptides applicable for the current study are shown. In the PRIDE repository more peptides are included which concern other projects.
