# Supplementary material for: Neurodegeneration and humoral response proteins in cerebrospinal fluid associate with pediatric-onset multiple sclerosis and not monophasic demyelinating syndromes in childhood
Source: Mult Scler. 2022 Sep 24;29(1):52–62. doi: 10.1177/13524585221125369 (PMC9896265; doi:10.1177/13524585221125369)
Supplement: sj-docx-3-msj-10.1177_13524585221125369 – Supplemental material for Neurodegeneration and humoral response proteins in cerebrospinal fluid associate with pediatric-onset multiple sclerosis and not monophasic demyelinating syndromes in childhood [file sj-docx-3-msj-10.1177_13524585221125369.docx]

**Supplementary file S3.** Table with patient characteristics of the samples used in the validation cohort separated for Dutch and Canadian samples.

|  | **VALIDATION** | | | | | |
| --- | --- | --- | --- | --- | --- | --- |
|  | **Dutch samples** | | | **Canadian samples** | | |
|  | **POMS**  n=34 | **mADS**  n=58 | **p value*** | **POMS**  n=14 | **mADS**  n=48 | **p value*** |
| **Sex**, no of females (%) | 19 (56) | 28 (48) | NS | 9 (64) | 21 (44) | NS |
| **Age at onset,** y, median [IQR] | 14.5 [13.4-15.9] | 5.8 [2.6-10.7] | **<0.001** | 14.0 [13.4-14.8] | 10.0 [5.9-12.4] | **<0.001** |
| **ADEM,** n (%) | 0 | 34 (59) | **<0.001** | 0 | 16 (33) | **0.013** |
| **Blood analyses**  - MOG-ab seropositive, n (%)  - AQP4-ab seropositive, n (%) | 0/29  0/17 | 15/54 (28)  0/35 | **0.002**  na | 0/14  0/14 | 17/48 (35)  0/48 | **0.007**  na |
| **CSF analyses**  - RBC, median [IQR]  - WBC, median [IQR]  - Total protein, median [IQR]  - Unique OCB, n (%) | 0.0 [0.0-9.0]  14.0 [5.0-28.0]  0.33 [0.25-0.38]  33/33 (100) | 0.0 [0.0-2.5]  8.0 [3.0-32.5]  0.28 [0.22-0.44]  6/42 (14) | NS  NS  NS  **<0.001** | 1.5 [0.0-6.3]  5.0 [2.0-10.2]  0.34 [0.31-0.43]  13/14 (93) | 1.0 [0-12.5]  9.0 [2.0-35.3]  0.27 [0.22-0.38]  4/40 (10) | NS  NS  NS  **<0.001** |
| **Time between disease onset and LP**, d, median [IQR] | 26.5 [10.0-56.8] | 9.0 [3.0-18.5] | **<0.001** | 11.0 [2.0-55.8] | 5.5 [3.0-13.3] | NS |
| **DMT use at time of LP** (%) | 0 | 0 | na | 0 | 0 | na |
| **FU,** m, median [IQR] | 47.5 [29.8-78.5] | 40.0 [18.0-93.3] | NS | 79 [45.3-132.3] | 82.5 [50.5-120.8] | NS |

*POMS vs. mADS using Chi-square (Fisher’s Exact) of Mann-Whitney U.

ADEM = acute disseminated encephalomyelitis, AQP4-ab = aquaporin-4 antibody, CSF = cerebrospinal fluid, DMT = disease modifying therapy, FU = follow-up, IQR = interquartile range, LP = lumbar puncture, mADS = monophasic acquired demyelinating syndrome, MOG-ab = myelin oligodendrocyte glycoprotein antibody, na = not applicable, OCB = oligoclonal bands, POMS = pediatric-onset multiple sclerosis, RBC = red blood cell, WBC = white blood cell.
