# Supplementary material for: Neurodegeneration and humoral response proteins in cerebrospinal fluid associate with pediatric-onset multiple sclerosis and not monophasic demyelinating syndromes in childhood
Source: Mult Scler. 2022 Sep 24;29(1):52–62. doi: 10.1177/13524585221125369 (PMC9896265; doi:10.1177/13524585221125369)
Supplement: sj-docx-4-msj-10.1177_13524585221125369 – Supplemental material for Neurodegeneration and humoral response proteins in cerebrospinal fluid associate with pediatric-onset multiple sclerosis and not monophasic demyelinating syndromes in childhood [file sj-docx-4-msj-10.1177_13524585221125369.docx]

**Supplementary file S4.** Table with correlation analyses PRM ratios and age.

| **Protein** | **Overall validation cohort** | | **POMS** | | **mADS** | | **mADS**  **non-ADEM** | | **mADS**  **ADEM** | |
| --- | --- | --- | --- | --- | --- | --- | --- | --- | --- | --- |
|  | Spearman’s ρ | P value | Spearman’s ρ | P value | Spearman’s ρ | P value | Spearman’s ρ | P value | Spearman’s ρ | P value |
| **SEMA7A** | 0.542 | **<0.001** | -0.009 | NS | 0.541 | **<0.001** | 0.386 | **0.003** | 0.207 | NS |
| **CPE** | 0.445 | **<0.001** | -0.114 | NS | 0.332 | **0.001** | 0.285 | **0.033** | 0.022 | NS |
| **MEGF8** | 0.562 | **<0.001** | -0.066 | NS | 0.531 | **<0.001** | 0.404 | **0.002** | 0.284 | **0.045** |
| **NEGR1** | 0.443 | **<0.001** | -0.094 | NS | 0.448 | **<0.001** | 0.284 | **0.034** | 0.248 | NS |
| **NUCB1** | 0.258 | **0.001** | -0.051 | NS | 0.203 | **0.037** | 0.233 | NS | -0.069 | NS |

ADEM = acute disseminated encephalomyelitis, CPE = Carboxypeptidase E, mADS = monophasic acquired demyelinating syndrome, MEGF8 = Multiple epidermal growth factor-like domains protein 8, NEGR1 = Neuronal growth regulator 1, NUCB1 = Nucleobindin-1, POMS = pediatric-onset multiple sclerosis, SEMA7A = Semaphorin-7A.
