# Supplementary material for: Neurodegeneration and humoral response proteins in cerebrospinal fluid associate with pediatric-onset multiple sclerosis and not monophasic demyelinating syndromes in childhood
Source: Mult Scler. 2022 Sep 24;29(1):52–62. doi: 10.1177/13524585221125369 (PMC9896265; doi:10.1177/13524585221125369)
Supplement: sj-docx-5-msj-10.1177_13524585221125369 – Supplemental material for Neurodegeneration and humoral response proteins in cerebrospinal fluid associate with pediatric-onset multiple sclerosis and not monophasic demyelinating syndromes in childhood [file sj-docx-5-msj-10.1177_13524585221125369.docx]

**Appendix**

**Members of the Dutch pediatric MS and ADEM study group:**

Dewi Bakker, MD, PhD (Amsterdam University Medical Center, Amsterdam); Maartje Boon (University Medical Center Groningen, Groningen); Rick Brandsma, MD, PhD (University Medical Center Utrecht, Utrecht); Kees Braun, MD, PhD (University Medical Center Utrecht, Utrecht); Coriene Catsman-Berrevoets, MD, PhD (Erasmus Medical Center/Sophia Children’s Hospital, Rotterdam); Katinke van Dijk, MD (Rijnstate Hospital, Arnhem); Judith Eikelenboom, MD, PhD (Dijklander Hospital, Hoorn); Marc Engelen, MD, PhD (Amsterdam University Medical Center, Amsterdam); Charlotte Haaxma, MD, PhD (Radboud University Medical Center, Nijmegen); A. Kurver, MD (Juliana Children’s Hospital/Haga Hospital, Den Haag); Jikke-Mien Niermeijer, MD, PhD (Elisabeth TweeSteden Hospital, Tilburg); Erik Niks, MD, PhD (Leiden University Medical Center, Leiden); Els Peeters, MD, PhD (Juliana Children’s Hospital/Haga Hospital, Den Haag); Cacha Peeters-Scholte, MD, PhD (Leiden University Medical Center, Leiden); Ruben Portier, MD (Medisch Spectrum Twente, Enschede); Johanneke de Rijk-van Andel, MD, PhD (Amphia Hospital, Breda); Johnny Samijn (Maasstad Hospital, Rotterdam), Herman Schippers, MD (St. Antonius Hospital, Utrecht); Lilian Sie, MD, PhD (Juliana Children’s Hospital/Haga Hospital, Den Haag); Irina Snoeck, MD (Juliana Children’s Hospital/Haga Hospital, Den Haag); Jeroen Vermeulen, MD, PhD (Maastricht University Medical Center, Maasstricht); Aad Verrips, MD, PhD (Canisius-Wilhelmina Hospital, Nijmegen); Frank Visscher, MD (Admiraal de Ruyter Hospital, Goes); Michel Willemsen, MD, PhD (Radboud University Medical Center, Nijmegen).

**Members of the Canadian Pediatric Demyelinating Disease Network:**

Principal Investigators: Brenda Banwell, MD (Children’s Hospital of Philadelphia, Perelman School of Medicine, University of Pennsylvania, Philadelphia PA); Amit Bar-Or, MD (Perelman School of Medicine, University of Pennsylvania, Philadelphia PA); Ruth Ann Marrie, MD (Max A Rady School of Medicine, University of Manitoba, Winnipeg, Manitoba); Douglas L. Arnold, MD (McGill University, Montreal Quebec); Ann E. Yeh, MD (Hospital for Sick Children, University of Toronto, Toronto, Ontario); Study Manager, Julia O’Mahony; Site Investigators: Mark Awuku, MD (University of Windsor, Windsor, Ontario); J. Burke Baird, MD (McMaster University, Hamilton, Ontario); Virender Bhan, MD (Dalhousie University, Halifax, Nova Scotia); David Buckley, MD (Janeway Children’s Health and Rehabilitation Centre, St John’s, Newfoundland and Labrador); David Callen, MD (Hamilton Health Sciences Center, Hamilton, Ontario); Mary B. Connolly, MBBCh (Children’s Hospital of British Columbia, Vancouver, British Columbia); Marie-Emmanuelle Dilenge, MD (Montreal Children’s Hospital, Montreal, Quebec); Asif Doja, MD (Children’s Hospital of Eastern Ontario, Ottawa, Ontario); Simon Levin, MD (University Hospital London, London, Ontario); Anne Lortie, MD (CHU Sainte-Justine, Montreal, Quebec); E. Athen MacDonald, MD (Hôtel-Dieu de Paris, Kingston, Ontario); Jean K. Mah, MD (Alberta Children’s Hospital, Calgary, Alberta); Brandon Meaney, MD (Hamilton Health Sciences Center, Hamilton, Ontario); David Meek, MD (St John Regional Hospital Facility, St John, New Brunswick); Daniela Pohl, MD (Children’s Hospital of Eastern Ontario, Ottawa, Ontario); Giullaume Sebire, MD (Montreal Children’s Hospital, Montreal, Quebec); Sunita Venkateswaran, MD (Children’s Hospital of Eastern Ontario, Ottawa, Ontario); Amy Waldman, MD (Children’s Hospital of Philadelphia, Philadelphia, Pennsylvania); Katherine Wambera, MD (Victoria General Hospital, Victoria, British Columbia); Ellen Wood, MD (Dalhousie University, Halifax, Nova Scotia); and Jerome Yager, MD (Children’s Stollery Hospital, Edmonton, Alberta).
